# Enhanced Ca^2+^ Entry Sustains the Activation of Akt in Glucose Deprived SH-SY5Y Cells

**DOI:** 10.3390/ijms23031386

**Published:** 2022-01-26

**Authors:** Maria Kourti, Danai Liaropoulou, Maria Paschou, Ioanna Giagklisi, Maria Paschalidi, Evangelia Petani, Panagiota Papazafiri

**Affiliations:** 1 Division of Animal and Human Physiology, Department of Biology, National and Kapodistrian University of Athens, Panepistimiopolis, 15784 Athens, Greece; mariakourti@med.uth.gr (M.K.); danaeliaropoulou@yahoo.com (D.L.); mapasch@biol.uoa.gr (M.P.); ioannag89@gmail.com (I.G.); maria__pas@hotmail.com (M.P.); e_petani@outlook.com.gr (E.P.); 2 Laboratory of Animal Physiology, Department of Biochemistry and Biotechnology, University of Thessaly, 41500 Larissa, Greece

**Keywords:** ischemia, hypoxia, glucose deprivation, Akt kinase, hypoxia-inducible factor 1, Ca^2+^ entry

## Abstract

The two crucial cellular insults that take place during cerebral ischemia are the loss of oxygen and loss of glucose, which can both activate a cascade of events leading to neuronal death. In addition, the toxic overactivation of neuronal excitatory receptors, leading to Ca^2+^ overload, may contribute to ischemic neuronal injury. Brain ischemia can be simulated in vitro by oxygen/glucose deprivation, which can be reversible by the re-establishment of physiological conditions. Accordingly, we examined the effects of glucose deprivation on the PI3K/Akt survival signaling pathway and its crosstalk with HIF-1α and Ca^2+^ homeostasis in SH-SY5Y human neuroblastoma cells. It was found that glucose withdrawal decreased HIF-1α protein levels even in the presence of the ischemia-mimicking CoCl_2._ On the contrary, and despite neuronal death, we identified a strong activation of the master pro-survival kinase Akt, a finding that was also confirmed by the increased phosphorylation of GSK3, a direct target of p-Akt. Remarkably, the elevated Ca^2+^ influx recorded was found to promptly trigger the activation of Akt, while a re-addition of glucose resulted in rapid restoration of both Ca^2+^ entry and p-Akt levels, highlighting the plasticity of neurons to respond to ischemic challenges and the important role of glucose homeostasis for multiple neurological disorders.

## 1. Introduction

Cerebral ischemia is a state of extreme metabolic stress occurring when the supply of oxygen and glucose is interrupted in a part of the brain, mainly due to the blockade of a cerebral artery [1]. Neuronal damage and ultimately death may result from the activation of signaling cascade pathways that are not yet fully understood [2,3]. To reduce damage from toxic insults, such as glutamate excitotoxicity and oxidative stress, neuronal cells have to deploy a set of molecular mechanisms allowing adaptation, if not restoration.

The heterodimeric hypoxia-inducible factor (HIF) is a transcription factor that is predominantly connected to the hypoxic status of cells and tissues mainly through its subunit HIF-1α [4], which includes one oxygen-dependent degradation domain that affects its stability [5]. In normoxic conditions, HIF-1α is ubiquitinated and degraded by the proteasome [5]. However, when intracellular oxygen levels are reduced, either in vivo or in vitro [6], or in the presence of various growth factors, oncogenes and cobalt chloride [7], the levels of HIF-1α are increased and HIF-1α translocates to the nucleus. There, it heterodimerizes with the constantly expressed HIF-1β subunit forming HIF-1 [5]. Extensive research has shown that HIF-1 regulates the transcription of at least 180 genes containing the 5′-(A/G)CGTG-3′motif, known as hypoxia-responsive element [5,8,9]. The vast majority of these genes are related to erythropoiesis, vascular reconstruction, metabolism, and cell survival [9,10]. Increased levels of both HIF-1 and its subunit HIF-1α are associated with the survival of cancer cells residing in the center of large and solid tumors, where they experience hypoxic or even anoxic conditions [5,11]. There, the reduced levels of oxygen results in the upregulation of HIF-1, and subsequently HIF-1α levels, which is responsible for the switch of cells metabolism from oxidative phosphorylation to anaerobic glycolysis and increase in glucose uptake [4,12]. Therefore, HIF-1 protects cells from any hypoxic-related impairments and helps them to adapt and survive in the hypoxic/ischemic environment [4,6].

One of the most prominent pro-survival signaling pathways is PI3K/Akt. Akt, a serine/threonine-specific protein kinase associated with the plasma membrane, is induced by the activation of phosphatidylinositol 3-kinase (PI3K), following stimulation of growth factor receptors with tyrosine kinase activity or G-protein-coupled receptors [13,14]. Once activated by phosphorylation, Akt translocates to the cytosol where it interacts with a plethora of substrates. The PI3K/Akt pathway is considered to mediate survival signals also in a wide range of neuronal cell types [15,16], while in one case, prolonged glucose deprivation induces Akt phosphorylation at Thr308 and the activation of its downstream targets, conferring cell survival under metabolic stress [17]. Inhibition of p-Akt in hypoxic-ischemic cortical neurons has a negative regulatory effect on HIF1-α, a key component of cellular response to brain ischemia [18]. As mentioned previously, HIF-1 is forcefully regulated by cellular oxygen availability [19,20]. However, in addition to oxygen, glucose also seems to have a modulating impact on HIF1-α levels, as reduced glucose availability results in decreased HIF-1α protein levels and expression of HIF1-dependent genes [21,22].

Recent studies suggest that the PI3K/Akt signaling pathway also possesses a primary role in intracellular Ca^2+^ homeostasis [23] and contributes to ischemic tolerance to sublethal oxygen and glucose deprivation in cortical neurons [24]. Ca^2+^ is a universal multifaceted second messenger able to encode cellular responses to a wide variety of external stimuli, thus playing a crucial role in neuronal signal transduction, regulation of neuronal excitability, and many cellular functions, such as gene transcription and cell proliferation [25,26]. Disturbances in neuronal Ca^2+^ homeostasis have been implicated in a variety of neuropathological conditions, including ischemia. In vitro ischemia, modeled by oxygen and glucose deprivation (OGD) in cultured neuronal cells, triggers cytosolic Ca^2+^ overload either due to Ca^2+^ influx through the plasma membrane, or by depletion of the intracellular Ca^2+^ stores. Particularly, the endoplasmic reticulum (ER) function has been shown to be disturbed during OGD conditions, whereas the dysfunction of SERCA, an ER-residing Ca^2+^ pump, represents an important mediator for ischemic Ca^2+^ overload and toxicity in various neuronal cell types, including cultured hippocampal neurons [27], isolated CA1 neurons [28], rat sensory neurons [29], and SH-SY5Y human neuroblastoma cells [30].

Herein, we discuss the involvement of PI3K/Akt during transient and prolonged ischemia, as well as during the restoration of glucose concentration in the human neuroblastoma cell line (SH-SY5Y) together with its crosstalk with HIF-1α and Ca^2+^. In addition to ER Ca^2+^ depletion, we show that glucose deprivation stimulates Ca^2+^ influx, which leads to a direct activation of Akt.

## 2. Results

Both glucose deprivation and oxygen withdrawal can affect cell function and fate. To assess their different impacts, we exposed SH-SY5Y cells to a glucose-free culture medium or the hypoxia-mimicking agent CoCl_2_.

### 2.1. Effect of Glucose Deprivation on p-Akt, p-GSK3α/β and HIF-1α Protein Levels

**Figure 1:**
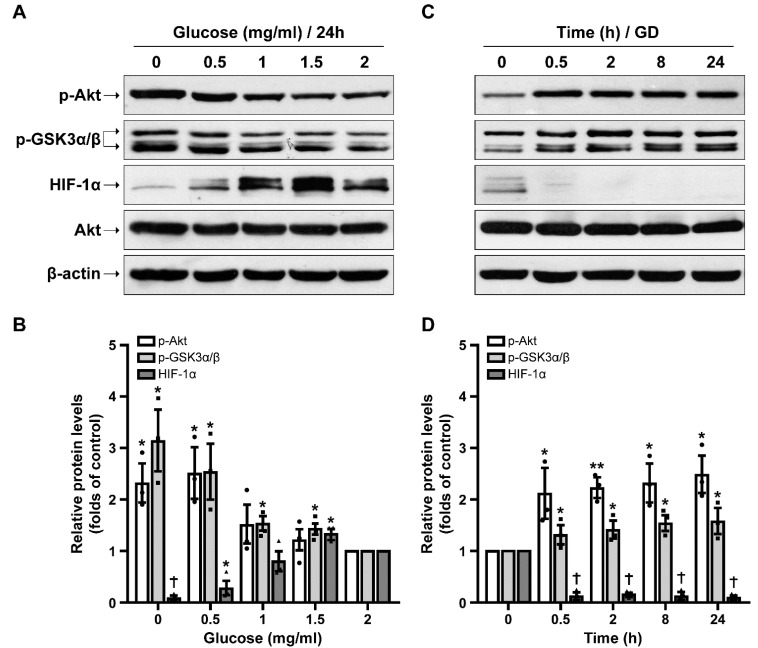
Opposite effects of glucose deprivation on p-Akt, p-GSK3α/β and HIF-1α protein levels. (**A**) Representative Western blot images of SH-SY5Y cells treated with increasing glucose concentrations (0–2 mg/mL in RPMI containing 10% FBS) for 24 h. (**B**) Quantification of p-Akt, p-GSK3α/β and HIF-1α protein levels for dose-dependent effect. Data are presented as mean ± S.E.M., *n* = 3, * *p* < 0.05, † *p* < 0.001, compared to control (2 mg/mL). (**C**) Representative Western blot images of cells incubated for 0.5–24 h in the absence of glucose (GD, glucose deprivation) in RPMI containing 10% FBS. (**D**) Quantification of p-Akt, p-GSK3α/β and HIF-1α protein levels for time-dependent effect. Data are presented as mean ± S.E.M., *n* = 3, * *p* < 0.05, ** *p* < 0.01, † *p* < 0.001, compared to control (0 h).

First, we examined the activation of the pro-survival signaling molecules Akt and GSK3α/β and the levels of HIF-1α, in SH-SY5Y cells exposed to increasing concentrations of glucose (0–2 mg/mL) for 24 h. Our results showed that activation of Akt is dose-dependent with low concentrations of glucose, and more specifically glucose deprivation (0 and 0.5 mg/mL) resulting in the highest induction of p-Akt levels, despite cell death (Figure 1A,B and Figure S1A). This effect was validated by the phosphorylation pattern of GSK3α/β, the downstream target of p-Akt, which was similarly influenced. In contrast, HIF-1α protein levels were reversely affected as glucose deprivation (0 and 0.5 mg/mL of glucose) led to a statistically significant decrease in HIF-1α levels compared to the control (2 mg/mL glucose), while higher concentrations resulted in an increase in the respective protein levels, reaching a peak at 1.5 mg/mL of glucose (Figure 1A,B). These results indicate that glucose deprivation has opposite effects on HIF-1α and p-Akt levels.

We further confirmed our findings by testing glucose deprivation effects in a time-dependent manner. In these experiments, p-Akt levels were immediately increased after the onset of glucose deprivation (glucose-free medium), reaching an almost 2.5-fold increase (Figure 1C,D). Similarly, GSK3α/β phosphorylation levels displayed the same pattern, although to a lesser extent. On the contrary, HIF-1α protein levels rapidly diminished after only 0.5 h of glucose deprivation and remained low throughout the 24 h incubation period.

### 2.2. Hypoxic Conditions Induced by CoCl_2_ Increases p-Akt, p-GSK3α/β and HIF-1a Protein Levels

Ischemia is characterized by the obtrusion of not only glucose availability, but also oxygen delivery, more commonly known as hypoxia. Here, we examined the responses of Akt, GSK3α/β, and HIF-1α by exposing the cells to CoCl_2_, a long-established artificial, hypoxia-mimicking agent.

**Figure 2:**
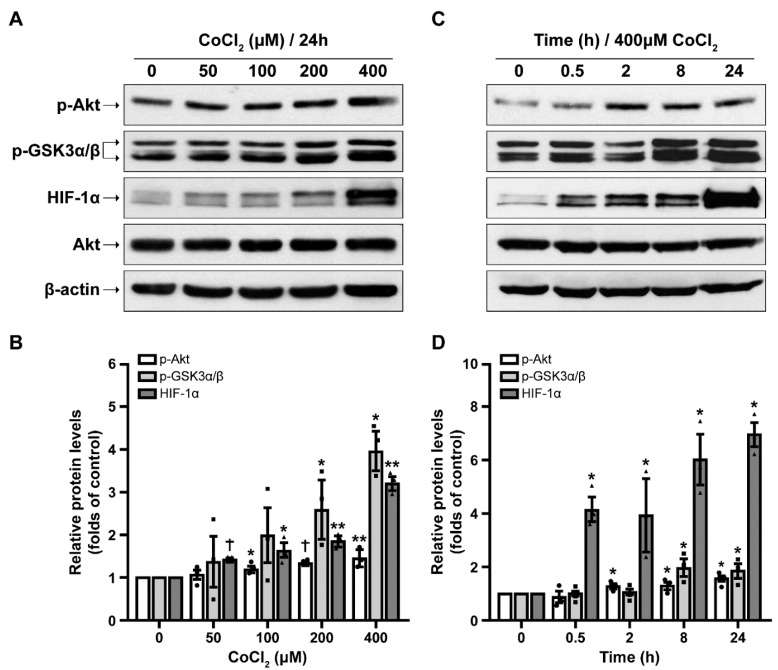
CoCl_2_ increases p-Akt, p-GSK3α/β and HIF-1α in a dose- and time- dependent manner. (**A**) Representative Western blot images of SH-SY5Y cells treated with increasing CoCl_2_ concentrations (50–400 μM) for 24 h. (**B**) Quantification of p-Akt, p-GSK3α/β and HIF-1α protein levels for dose–dependent effect. (**C**) Representative Western blot images of cells treated with 400 μΜ CoCl_2_ for 0.5–24 h. (**D**) Quantification of p-Akt, p-GSK3α/β and HIF-1α protein levels for time–dependent effect. Data are presented as mean ± S.E.M., *n* = 3, * p < 0.05, ** *p* < 0.01, † *p* < 0.001 compared to control (0 μΜ and 0 h for (**C**,**D**) respectively).

Our results clearly illustrate that increasing concentrations of CoCl_2_ (from 50 up to 400 μM) resulted in a significant induction of Akt and GSK3α/β phosphorylation in the span of 24 h (Figure 2A,B). As expected, HIF-1α levels already increased in the presence of only 50 μΜ CoCl_2_.

We further confirmed our findings in time-course responses and in the presence of 400 μM CoCl_2_. As shown in Figure 2C,D, p-Akt and p-GSK3α/β levels gradually rose after exposure of cells to CoCl_2_ for longer incubation periods (0.5, 2, 8, and 24 h). Moreover, HIF-1α levels were found to be drastically increased by four-fold compared to control conditions (0 h) already in 0.5 h of incubation with CoCl_2_, and gradually peaked at 24 h.

So far, our results indicate that glucose deprivation has a strong and opposite impact on Akt phosphorylation and HIF-1α, while hypoxia influences mostly HIF-1α levels. However, since CoCl_2_ also causes a moderate increase in p-Akt levels, it could be assumed that these two proteins may be associated. It is already known that Akt and HIF-1α crosstalk, especially in cancer, resulting in combined or enhanced effects [31,32]. To attest to this crosstalk, cells were pre-treated for 30 min with 100 nM wortmannin, a PI3K/Akt-specific inhibitor, and subsequently incubated in a complete medium containing 400 μM CoCl_2_ or in a medium without glucose (0 mg/mL) for 4 h. CoCl_2_ and glucose deprivation elicited the expected effects on p-Akt and HIF-1α levels, while wortmannin almost completely inhibited Akt phosphorylation (Figure S2A). In addition, PI3K/Akt inhibition mitigated the response of all three proteins studied under hypoxia and glucose deprivation conditions, indicating a correlation of Akt phosphorylation and HIF-1α protein levels.

Next, to investigate whether glucose deprivation leads to HIF-1α proteasome degradation, cells were treated with 10 μM MG132, a proteasome specific inhibitor, for 4 h. No significant changes in HIF-1α levels were detected after pre-treatment with MG132, either in the presence or absence of glucose (Figure S2B). Therefore, we conclude that the observed fluctuations in HIF-1α levels were not due to proteasome-mediated degradation.

### 2.3. Combined Effect of CoCl_2_ and Glucose Deprivation on p-Akt, and HIF-1a Protein Levels

**Figure 3:**
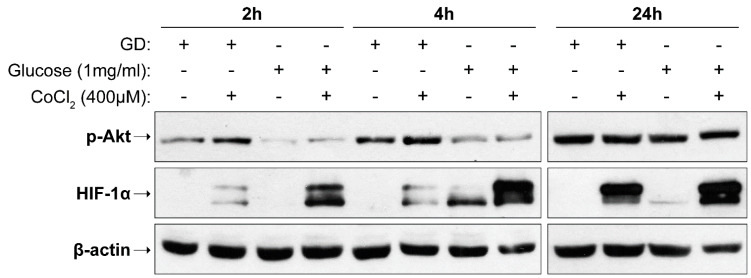
Glucose deprivation decreases HIF-1α levels during hypoxia. Representative Western blot images of p-Akt and HIF-1α protein levels (from two separate experiments) in SH-SY5Y cells incubated for 2, 4, and 24 h in the absence of glucose (GD, glucose deprivation), or in the presence of 1 mg/mL glucose, either without or with CoCl_2_.

For better simulation of ischemic conditions, cells were cultured in a glucose-free medium (containing 10% FBS) supplemented with 400 μΜ CoCl_2_ or 1 mg/mL glucose for short (2, 4 h) and long (24 h) incubation periods. Interestingly, the combined action of CoCl_2_ and glucose deprivation also caused the activation of Akt (Figure 3). This result verifies the unexpected increase in Akt phosphorylation observed in each condition alone (and glucose deprivation).

Additionally, glucose deprivation attenuated HIF-1α levels in all incubation periods examined, even in the presence of CoCl_2_. Of note, the effect of glucose withdrawal was more pronounced during short-term treatments.

### 2.4. Re-Administration of Glucose Restores p-Akt Levels

**Figure 4:**
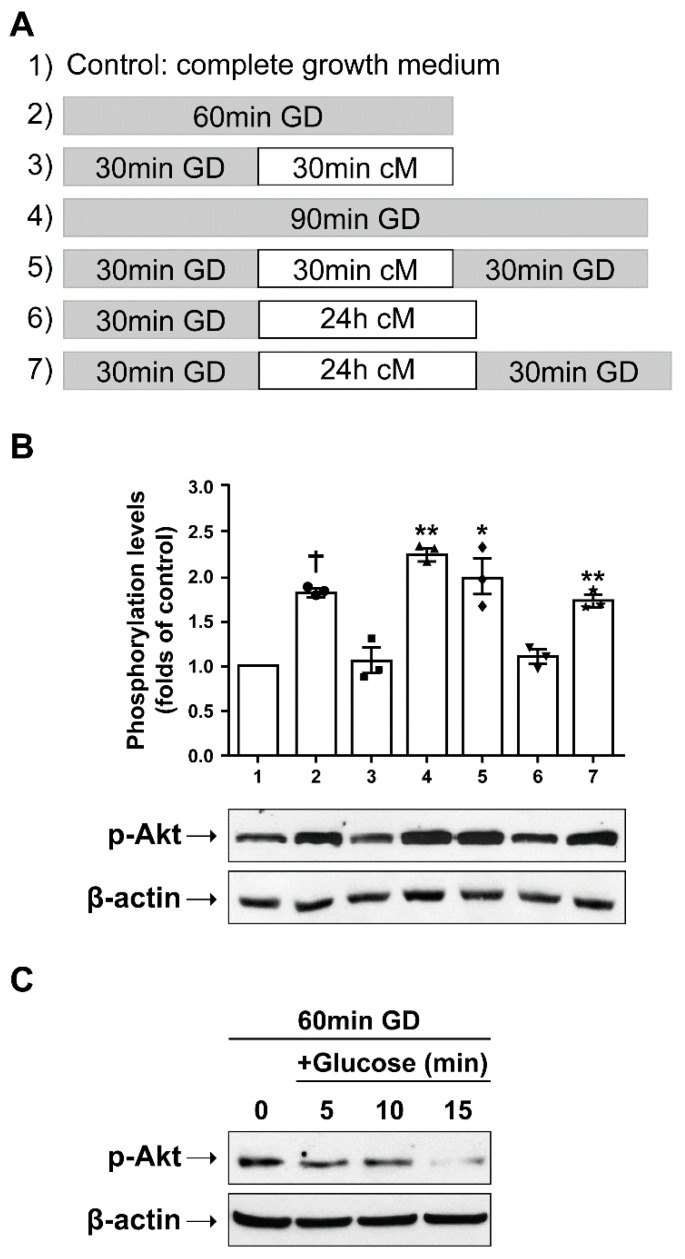
Glucose deprivation–induced Akt phosphorylation is partially restored after the glucose re-addition. (**A**) Protocol of repetitive periods of glucose deprivation using a glucose-free medium and glucose re-addition using a conditioned medium (cM). Control cells were incubated in a complete growth medium. (**B**) Representative Western blot images and quantification of p-Akt levels in SH-SY5Y cells incubated as shown in (**A**), compared to control cells. Data are presented as mean ± S.E.M., *n* = 3, * *p* < 0.05, ** *p* < 0.01, † *p* < 0.001, compared to control. (**C**) Representative Western blot image of p-Akt in glucose-deprived SH-SY5Y cells (for 60 min) followed by glucose supplementation (1 mg/mL) for 5, 10, and 15 min.

Subsequently, we examined whether glucose re-addition in the culture medium could reverse the activation of Akt. To accomplish this, the conditioned medium (cM) from SH-SY5Y cells was collected when the cells were at 70–80% confluency and was added to SH-SY5Y cells that were glucose deprived for 30 min. The duration of the cM incubation period combined with glucose deprivation intervals varied and is depicted in Figure 4A.

This set of experiments showed that glucose deprivation elicits a statistically significant increase in p-Akt levels, and that re-addition of glucose (substitution of culture medium with cM) could conditionally counter-balance this response (Figure 4B). Akt phosphorylation appears to be highly sensitive to cM components, including glucose, the presence of which is apparently one of the most important parameters influencing p-Akt levels at the time of cell harvesting. Noticeably, p-Akt levels were found to be increased each time cells were collected immediately from the glucose deprivation condition, regardless of what had preceded. However, this increase is readily reversed in the presence of glucose for as little as 5 min (Figure 4C).

### 2.5. Ca^2+^ Measurements

**Figure 5:**
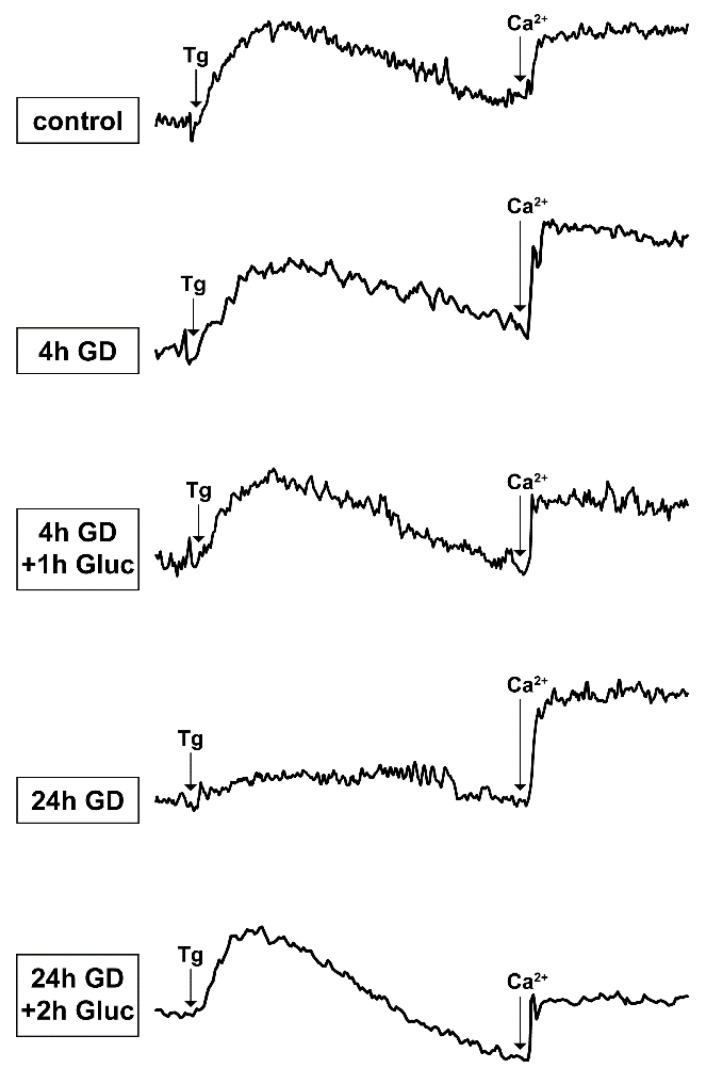
Reversal of changes in Ca^2+^ homeostasis caused by short- and long-term glucose deprivation (GD) upon the re-addition of glucose. ER Ca^2+^ content was measured upon stimulation of cells with Tg in Ca^2+^ free-KRH and SOCE was measured by addition of 3 mM CaCl_2_ (arrows). Glucose deprivation of SH-SY5Y cells lasted 4 and 24 h and glucose restoration for 1 and 2 h, respectively. Representative traces from 4–6 independent experiments.

Excessive mobilization of Ca^2+^ is considered to contribute to ischemic neuronal damage. Ca^2+^ is derived from extracellular or intracellular routes, namely, the ER. It is well established that depletion of the ER from Ca^2+^ induces a subsequent, store-operated channel (SOC)-mediated Ca^2+^ influx from the extracellular environment. In order to assess Ca^2+^ mobilization during glucose deprivation, Fura-2 AM loaded cells were challenged with 100 nΜ Thapsigargin (Tg). Tg is an irreversible inhibitor of the SERCA pumps, which triggers the depletion of the ER from Ca^2+^ ions, and is thus, widely used to measure ER Ca^2+^ content [33]. ER Ca^2+^ depletion, in turn, results in the subsequent stimulation of store operated Ca^2+^ entry (SOCE), which can be evaluated upon the addition of 3 mΜ CaCl_2_. In this set of measurements, we observed that glucose deprivation for either 4 or 24 h leads to increased basal levels of [Ca^2+^]_i_, reduced ER Ca^2+^ content, and increased SOCE (Table 1, Figure 5). Surprisingly, although the Tg-induced response was almost completely abolished after 24 h of glucose deprivation, the re-addition of glucose for only 2 h restored the ER Ca^2+^ content. Regarding SOCE, we found that 4 h and 24 h of glucose deprivation induced a significant increase in Ca^2+^ entry, which was also restored to control levels after the re-addition of glucose.

### 2.6. Ca^2+^-Induced Modulation of PI3K/Akt and HIF-1α

**Figure 6:**
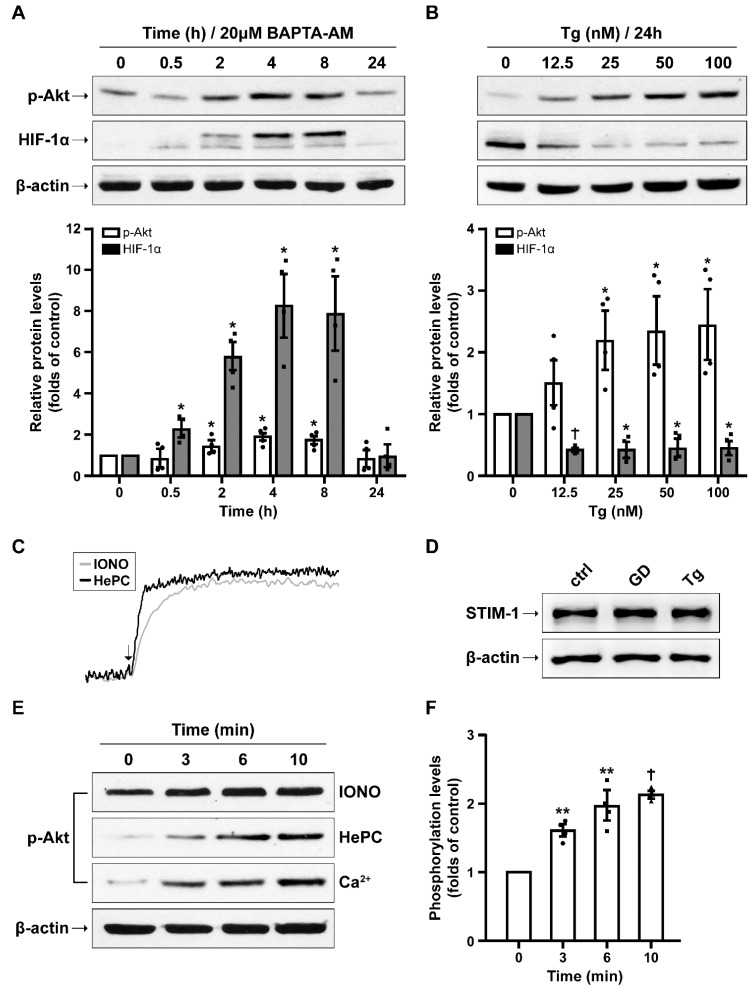
Ca^2+^ entry activates Akt. (**A**) Representative Western blot images and quantification of p-Akt and HIF-1α levels in SH-SY5Y cells incubated with 20 μΜ BAPTA-AM, a Ca^2+^ chelating agent, for 0.5 up to 24 h. (**B**) Representative Western blot images and quantification of p-Akt, and HIF-1α levels in SH-SY5Y cells treated with thapsigargin (Tg, 0–100 nM) for 24 h. (**C**) Ca^2+^ entry induced by ionomycin (IONO) or miltefosine (HePC) measured in Fura-2 loaded SH-SY5Y cells in Ca^2+^ containing KRH (representative traces from 3–6 independent experiments). (**D**) Representative Western blot image of STIM1 levels during glucose deprivation (GD) or Tg (100 nM) treatment for 24 h. (**E**,**F**) Representative Western blot images and quantification of p-Akt levels in SH-SY5Y cells detached and re-suspended in KRH buffer supplemented with IONO (500 nM) or HePC (20 μM). In the case of CaCl_2_ (3 mM), cells were pre-treated with Tg in Ca^2+^ free-KRH for 10 min. Cells were harvested after 0, 3, 6, and 10 min of incubation. All data are presented as mean ± S.E.M., *n* = 4, * *p* < 0.05, ** *p* < 0.01, † *p* < 0.001, compared to controls.

We have previously shown that Ca^2+^ mobilization affects both the p-Akt and HIF-1α levels in cancer epithelial cells [34]. Hence, we questioned whether this crosstalk is also taking place in SH-SY5Y cells. For this, cells were treated with Ca^2+^ chelating or mobilizing agents. First, we examined the effect of BAPTA-AM, a Ca^2+^ chelating agent, and our findings revealed that HIF-1α and p-Akt levels increased as soon as 30 min after a 20 μΜ BAPTA-AM addition, reaching a peak at 8 h (Figure 6A). However, prolonged incubation with BAPTA-AM is known to have toxic effects through the inhibition of protein translation, and this is the most probable reason why these proteins steeply diminish at 24 h of incubation. This result indicates that a decrease in cytosolic Ca^2+^ favors the increase of both HIF-1α and p-Akt.

Next, we used thapsigargin (Tg) to simulate ER Ca^2+^ depletion, which was found to accompany glucose deprivation (Figure 5). Cells were incubated in the presence of increasing concentrations of Tg (0–100 nM) for 24 h, and our results showed a gradual increase in p-Akt in contrast to HIF-1α levels, which were drastically diminished. (Figure 6B). Of note, the profile of changes induced by Tg was impressively similar to that of glucose deprivation. Interestingly, the same pattern was detected in primary neuronal cultures, indicating a common underlying mechanism (Figure S3). Upon ER Ca^2+^ depletion, the major sensor of ER Ca^2+^ content and critical partner of store-operated Ca^2+^ entry, STIM1 (Stromal interaction molecule 1), moves close to the plasma membrane and facilitates Ca^2+^ entry through the ORAI1 component of the store-operated channel. We noticed unchanged levels of STIM1 after glucose deprivation and Tg treatment for 24 h (Figure 6D), demonstrating that the location or activity of both or either ORAI1 or STIM1 could be altered.

Finally, we sought to address whether Ca^2+^ entry, immediate or subsequent to ER Ca^2+^ depletion, could account for the increase in p-Akt levels observed during glucose deprivation. For this, we used cells re-suspended in KRH solution containing 3 mM CaCl_2_ and then incubated either with ionomycin (IONO) or miltefosine (HePC) for 0, 3, 6, or 10 min (Figure 6E,F). IONO and HePC are factors that instantly trigger massive Ca^2+^ mobilization (Figure 6C) and were used to examine the effect of early Ca^2+^ entry on the Akt phosphorylation levels. As a final point, we examined whether the stimulation of SOCE could also activate Akt. Cell suspension in the KRH solution devoid of CaCl_2_ was pre-incubated in the presence of Tg for 10 min, and samples were collected at 0, 3, 6, and 10 min after Ca^2+^ re-addition. All the above experimental procedures showed that p-Akt levels increased rapidly upon plasma membrane Ca^2+^ entry (Figure 6E,F).

## 3. Discussion

Reduced brain oxygen and glucose transport under ischemic conditions causes energy deficiency, which leads to oxidative stress, inflammation, blood-brain barrier dysfunction, and eventually, cell death. Unless blood circulation is restored rapidly, tissue necrosis begins from the center of the occluded area, which spreads completely within one to three days and affects all cell types [35]. Areas of the brain such as the cerebral cortex, cerebellum, hypothalamus, and hippocampus are extremely vulnerable to ischemic conditions. Severe hippocampal damage results in difficulty forming new memories and often affects memories created before the ischemic attack. In addition, Purkinje cells in the cerebellum are particularly sensitive to such conditions [36]. However, there is a significant heterogeneity in the ability of different brain cell types, even cells of the same type located in distinct regions, to cope with focal ischemia. Thus, neurons and oligodendrocytes [37] are the most vulnerable to ischemia, while most astrocytes are less prone to these conditions, due to better maintenance of their energy metabolism [38,39]. Consequently, cells have to employ numerous different mechanisms in order to adapt and function in hypoxic conditions.

In the present study, the response of SH-SY5Y neuroblastoma cells was investigated mainly under ischemia-like glucose deprivation conditions. Because much of the neuronal damage associated with cerebral ischemia is delayed for hours or even days after the initial attack, it is likely that immediate intervention after the onset of ischemia may prevent or alleviate the injury. For this reason, we also examined the effect of the short recovery of glucose levels, in an attempt to restore physiological conditions.

The hypoxia-inducible factor 1 (HIF-1), and particularly its subunit HIF-1α, is the master regulator of cellular adaptation in a low oxygen environment. HIF-1α is required for cellular protection and metabolic alterations and, as such, is considered one of the many downstream targets of Akt. We and several other groups have established that under conditions that promote cell survival, there is a dose-dependent increase in both the protein levels of HIF-1α and p-Akt [34,40,41,42]. In addition, previous results of our group indicate that a partial connection between these two proteins takes place even under specific stressful conditions, i.e., in the presence of the carcinogenic benzo [α] pyrene [42]. Here, a similar connection was also proved both during glucose deprivation and in the presence of CoCl_2._ This is particularly significant in glucose deprivation conditions, during which Akt could maintain HIF-1α levels to a minimum, counteracting its drastic decrease in this condition.

The regulation of HIF-1α levels is also connected to Ca^2+^ signaling, as highlighted by a number of studies [43,44,45]. We have previously shown that chelation of cytosolic Ca^2+^ by BAPTA-AM leads to increased protein levels of HIF-1α [34], a finding that was also confirmed in the present study using SH-SY5Y cells. This significant increase in HIF-1α levels was suggested to rely on inhibition of PHD2 (prolyl hydroxylase domain-2) [46], thus preventing its degradation in the proteasome, but total cellular Ca^2+^ levels were not affected during the hypoxic conditions. Moreover, it has been observed that cilnidipine, a Ca^2+^ channel blocker, repressed the synthesis of the HIF-1α protein by inhibiting the activity of Akt, in non-neuronal cells [47]. However, this inhibition was again not dependent on Ca^2+^ homeostasis changes. Thus, although most studies indicate that low Ca^2+^ concentrations favor HIF-1α stability, this type of regulation needs to be explored in more detail.

It is well known that ischemia causes ER stress, consequently leading to an imbalance between survival and death signals and Ca^2+^ influx from the extracellular space [30]. Ca^2+^ entry is activated by the depletion of Ca^2+^ from the ER and mediated by STIM1, which moves close to the plasma membrane and forms highly Ca^2+^ selective pores with ORAI1. This leads to Ca^2+^ influx from the extracellular space directly into the ER through the Ca^2+^ release-activated Ca^2+^ current (I_CRAC_) [48,49]. Even though ER Ca^2+^ depletion represents an undisputed factor of neuronal cell injury, the modulation of the STIM1/ORAI1 complex during ischemia is still under scrutiny. For instance, ORAI1 and STIM1 levels are significantly reduced in hypoxic cortical neurons [50], whereas STIM1 expression is elevated in hippocampal neurons exposed to chemically induced hypoxia [51]. We found that STIM1 levels remain unchanged after long-term incubation with Tg or in the absence of glucose, i.e., when ER is depleted and Ca^2+^ entry is stimulated. We consider that either the localization or activity of the STIM1/ORAI1 complex is more susceptible to changes, and further studies are needed in this direction.

Apart from low oxygen levels, hypoglycemia is another major feature of ischemia. We found that HIF-1α and Akt are differentially affected by these two components. Specifically, HIF-1α is increased in simulated hypoxic conditions but is drastically decreased in the absence of glucose. Although the correlation between glucose and HIF-1α is anticipated, Akt, on the contrary, is activated in both conditions in spite of cell death. Other studies also present evidence that activation of Akt accompanies cell death [52], and a role in resistance to ER stress is attributed to Akt [53]. However, a direct link between ER-induced Ca^2+^ entry and Akt activation has not been established yet. The immediate and sustained Ca^2+^ influx at early times after West Nile virus infection was found to play a predominant role in cell signaling, and specifically in the activation of Akt [54]. Aligned with the above, we found a rapid activation of Akt in cell suspensions deprived of growth factors upon stimulation of Ca^2+^ entry. This type of activation could also explain the increase in p-Akt that we observed in the presence of BAPTA-AM, contrary to the one we found in our previous work on epithelial cells [34]. Possibly, the presence of BAPTA-AM in SH-SY5Y cells, causes a shift in the balance of Ca^2+^ between the ER and the cytosol, leading to ER Ca^2+^ depletion and subsequent Ca^2+^ influx. Hence, local elementary events of Ca^2+^ mobilization, such as SOCE, seem to rigorously influence the mechanisms of cellular homeostasis by fine tuning ER Ca^2+^ stores for cellular signaling and function.

As mentioned above, a key mediator of the SOCE mechanism is the plasma membrane channel ORAI1. Recently, it has been shown that in the presence of PI3K inhibitors, the abundance of ORAI1 at the cell’s surface is restricted [55]. Additionally, it is reported that specific Ca^2+^ channels in lipid rafts comprise important sites linking Ca^2+^ entry directly to Akt signaling [56], a phenomenon that could result from the hyper-activation of store-operated receptors. Based on the above, and on our results, we might suggest that, under specific conditions, PI3K/Akt activation and Ca^2+^ entry form a mutually serving functional loop. In the latter, Ca^2+^ entry leads to the activation of PI3K/Akt, which is then engaged to support Ca^2+^ channel trafficking and is not accessible to downstream cell survival pathways.

In summary, we have demonstrated that glucose deprivation, a component of ischemia, activates Ca^2+^ entry in a reversible manner. This Ca^2+^ entry plays a primordial role in replenishing the depleted Ca^2+^ ER while it also sustains the activation of Akt. Such Ca^2+^ fluctuations, aside from which proteins are regulated, may play a key role in the pathophysiology of ageing, diabetes, and a broad range of neurological diseases. As timely and effective restoration of ER Ca^2+^ content could represent a potential treatment for neurodegeneration, these findings may prove to be useful not only for the prevention of stroke progression but also for neurodegenerative diseases with underlying Ca^2+^ disorders.

## 4. Materials and Methods

### 4.1. Cell Culture and Treatments

SH-SY5Y human neuroblastoma cells (CRL-2266, obtained from the American Type Culture Collection, Rockville, MD, USA) were maintained in RPMI 1640 medium (Gibco, Grand Island, NY, USA) supplemented with 10% fetal bovine serum (FBS), 100 U/mL penicillin, 100 μg/mL streptomycin (PAA Laboratories, Pasching, Austria) at 37 °C in a humidified atmosphere of 5% CO_2_. Hypoxic conditions were simulated by adding CoCl_2_ (50–400 μΜ, Sigma-Aldrich, St. Louis, MO, USA) in culture medium and hypoglycaemic conditions were simulated by glucose-free RPMI medium supplemented with 10% FBS. Depletion of ER was induced by thapsigargin (Tg; 12.5–100 nM, Sigma-Aldrich), an inhibitor of SERCA pump. Wortmannin (100 nM, Sigma-Aldrich) was used as a specific inhibitor of PI3K/Akt and MG132 (10 μΜ, Calbiochem, San Diego, CA, USA) as a proteasome inhibitor.

For a limited number of experiments, primary neuronal cultures were used. Briefly, pregnant C57BL/6 mice, were sacrificed by cervical dislocation and fetuses (embryonic day 16–18) were removed under sterile conditions, placed in ice cold PBS, and kept on ice for microscopic dissection. Cortices were minced and digested with 0.125% trypsin (Sigma-Aldrich, St. Louis, MO, USA) in calcium, magnesium-free PBS containing 5 μg/mL DNAse at 37 °C for 20 min. Cells were suspended in Neurobasal medium (Gibco BRL, Grand Island, NY, USA), plated in 35 mm plates (Greiner Bio-One, Cellstar, Kremsmünster, Austria) at a density of 5 × 10^5^ cells and incubated at 37 °C in a humidified atmosphere of 5% CO_2_. All animal procedures were carried out according to the Greek Law 56/2013, in conformity with European Union guidelines.

### 4.2. Preparation of Total Protein Extracts and Western Blot Analysis

For Western blot experiments, SH-SY5Y cells were washed twice with ice-cold PBS, harvested by scraping, lysed in ice-cold Buffer G containing 20 mM β-glycerophosphate, 20 mM NaF, 2 mM EDTA, 0.2 mM Na_3_VO_4_, 10 mM benzamidine, 20 mM HEPES, pH 7.5, supplemented with 0.5% (*v*/*v*) Triton X-100 and a mixture of protease inhibitors (200 μΜ leupeptin, 5 mM DTT, 300 μΜ PMSF, and 10 μΜ E64) and incubated on ice for 30 min. Lysates were then centrifuged at 10,000× *g* for 10 min and protein concentration in the supernatant was determined by the Bradford assay (Bio-Rad). Equal amounts of total protein (50 μg protein per sample) were separated in 8% (*w*/*v*) SDS-PAGE gels and blotted onto nitrocellulose membranes (Porablot NCP; Macherey-Nagel, Düren, Germany). Membranes were incubated overnight at 4 °C with the respective primary antibodies (Table S1) and proteins were detected using the enhanced chemiluminescence’s reaction (ECL, Thermo Scientific, NH, USA) onto a X-OMAT AR film or in an 8800 FluorChem Imaging System (Alpha Innotech Corp., San Leandro, CA, USA). The intensity of each immunoreactive band was estimated by densitometric quantification using the ImageJ Software (https://imagej.nih.gov/ij; NIH, Bethesda, MD, USA). The levels of the phosphorylated proteins were expressed as the ratio of the pixel intensity of the phosphorylated protein to the intensity of the total protein in each case. In all cases, β-actin was used as an internal loading control.

### 4.3. Cytosolic Ca^2+^ Measurement

Intracellular Ca^2+^ concentration ([Ca^2+^]_i_) was measured in SH-SY5Y cells using the Ca^2+^-sensitive dye, Fura-2. Briefly, cells were detached, centrifuged, and resuspended in complete Krebs–Ringer–Henseleit (KRH) buffer (125 mM NaCl, 5 mM KCl, 1.2 mM MgSO_4_, 1.2 mM KH_2_PO_4_, 25 mM Hepes/NaOH, 2 mM CaCl_2_, 6 mM glucose) containing 50 μΜ DTPA. Afterwards, cells (10 × 10^6^ cells/mL) were loaded with 5 μM Fura-2 AM (Calbiochem, CA, USA) in KRH containing 200 μΜ sulfinpyrazone and incubated at 37 °C for 30 min. After washing, cells were centrifuged and resuspended in KRH buffer (1 × 10^6^ cells/sample) or in KRH without glucose in case of simulating ischemic conditions. Cell aliquots were transferred to a thermostatted cuvette in a Perkin Elmer LS-50 fluorometer (Norwalk, CT, USA) and maintained at 37 °C, under continuous stirring. Fura-2 fluorescence was monitored with excitation and emission wavelengths of 340 nm and 510 nm respectively. Ca^2+^ entry was measured in cell samples resuspended in a Ca^2+^-free KRH containing 10 μM EGTA. Thapsigargin, the sarcoplasmic-endoplasmic reticulum Ca^2+^-ATPase blocker, was added to induce depletion of intracellular Ca^2+^ stores. The recording was continued for up to 10 min until the end of the first peak and Ca^2+^, at a final concentration of 3mM, was then added. Traces were recorded and analysed as previously described [57] using the equation [Ca^2+^]_i_ = K_d_ × (F − F_min_)/(F_max_ − F), where K_d_ is the dissociation constant of the Fura-2–Ca^2+^ complex that was assumed to be 225 nM. The minimum values (F_min_) were determined by an intrinsic calibration procedure after the addition of 3 mM EGTA whereas, to estimate the maximal values (F_max_), CaCl_2_ (13 mM) was added after cell lysis with 0.1% Triton X-100. Changes in [Ca^2+^]_i_ were determined by measuring peak or plateau values and expressing them as the change from basal levels.

### 4.4. Cell Viability Assay

SH-SY5Y cells (3 × 10^4^ cells/well) were seeded into 96-well plates and cultured in RPMI 1640 medium for 24 h at 37 °C. Afterwards, cells were treated with increasing concentrations of CoCl_2_ (6.25–400 μM), glucose (0–2 mg/mL) in RPMI containing 10% FBS for 24 h. Cell viability was determined using the MTT colorimetric method. Briefly, at the end of the incubation period 2.5 mg/mL MTT (Applichem, Darmstadt, Germany) solution was added to the culture medium and cells were incubated at 37 °C for 4 h. Afterwards, the cultured medium was removed and 100 μL of DMSO was added to each well to dissolve the formazan crystals. The absorbance of each sample was measured at 570 nm in an ELISA plate reader (Denley, West Sussex, UK). Cell viability was expressed as the percentage of the absorbance value compared to control cells.

### 4.5. Data Analysis

Protein levels were normalized using β-actin or GAPDH as internal loading controls. In each experiment, the pixel intensity values of the examined proteins of treated samples (ratio of values of protein of interest to values of loading control) were expressed as fold changes of control samples (set at 1).

All data shown are representative of at least three independent experiments and are presented as mean ± standard error of the mean (S.E.M.). Statistical significance of differences was evaluated using one-way ANOVA, followed by Dunnett’s multiple comparison test. Probability (*p*) values < 0.05 were considered significant. Statistical analysis was performed using the GraphPad Prism software (version 8.0.0; San Diego, CA, USA).

## Figures and Tables

**Table 1:** Effect of glucose deprivation on intracellular Ca^2+^ concentration ([Ca^2+^]_i_), ER Ca^2+^ content and Ca^2+^ entry.

Cell Culture Condition	[Ca^2+^]_i_, nM	ER Ca^2+^ Content (% [Ca^2+^]_i_)	Ca^2+^ Entry (% of ER Ca^2+^ Content)
Control	47.8 ± 4	297.9 ± 21	71.8 ± 8
GD 4 h	64.3 ± 8 *	107.5 ± 14 **	98.3 ± 6 **
GD 4 h & 1 h Gluc	62.5 ± 8 *	146.8 ± 12 **^‡^	82.4 ± 5
GD 24 h	83.2 ± 12 *	23.6 ± 2 ^†^	482.6 ± 3 ^†^
GD 24 h & 2 h Gluc	78.9 ± 6 *	106.4 ± 12 ^‡^	66.7 ± 9 ^‡^

Values shown are means ± S.E.M. from 4–6 independent experiments. * p < 0.05, ** *p* < 0.01, ^†^ *p* < 0.001 compared to control, **^‡^** *p* < 0.001 compared to the corresponding GD sample.

## Data Availability

Not applicable.

## Supplementary Materials

The following supporting information can be downloaded at: https://www.mdpi.com/article/10.3390/ijms23031386/s1.
