# Supplementary material for: Enhanced Ca2+ Entry Sustains the Activation of Akt in Glucose Deprived SH-SY5Y Cells
Source: Int J Mol Sci. 2022 Jan 26;23(3):1386. doi: 10.3390/ijms23031386 (PMC8835965; doi:10.3390/ijms23031386)

Antibody	Supplier	Cat. Number
p-Akt (Ser 473)	Cell Signaling Technology	#9271
p-GSK3α/β (Ser21/9)	Cell Signaling Technology	#8566
HIF-1α	BD Transduction Laboratories	610959
Akt	Cell Signaling Technology	#4685
β-Actin	Sigma-Aldrich	A2228
STIM1	Cell Signaling Technology	#5668
p-ERK1/2	Cell Signaling Technology	#9101
GAPDH	Santa Cruz Biotechnology	365062
Anti-mouse conjugated with HRP	Millipore	AP130P
Anti-rabbit conjugated with HRP	Jackson ImmunoResearch	111-035-144

Table S1. Primary antibodies used in western blot analysis.

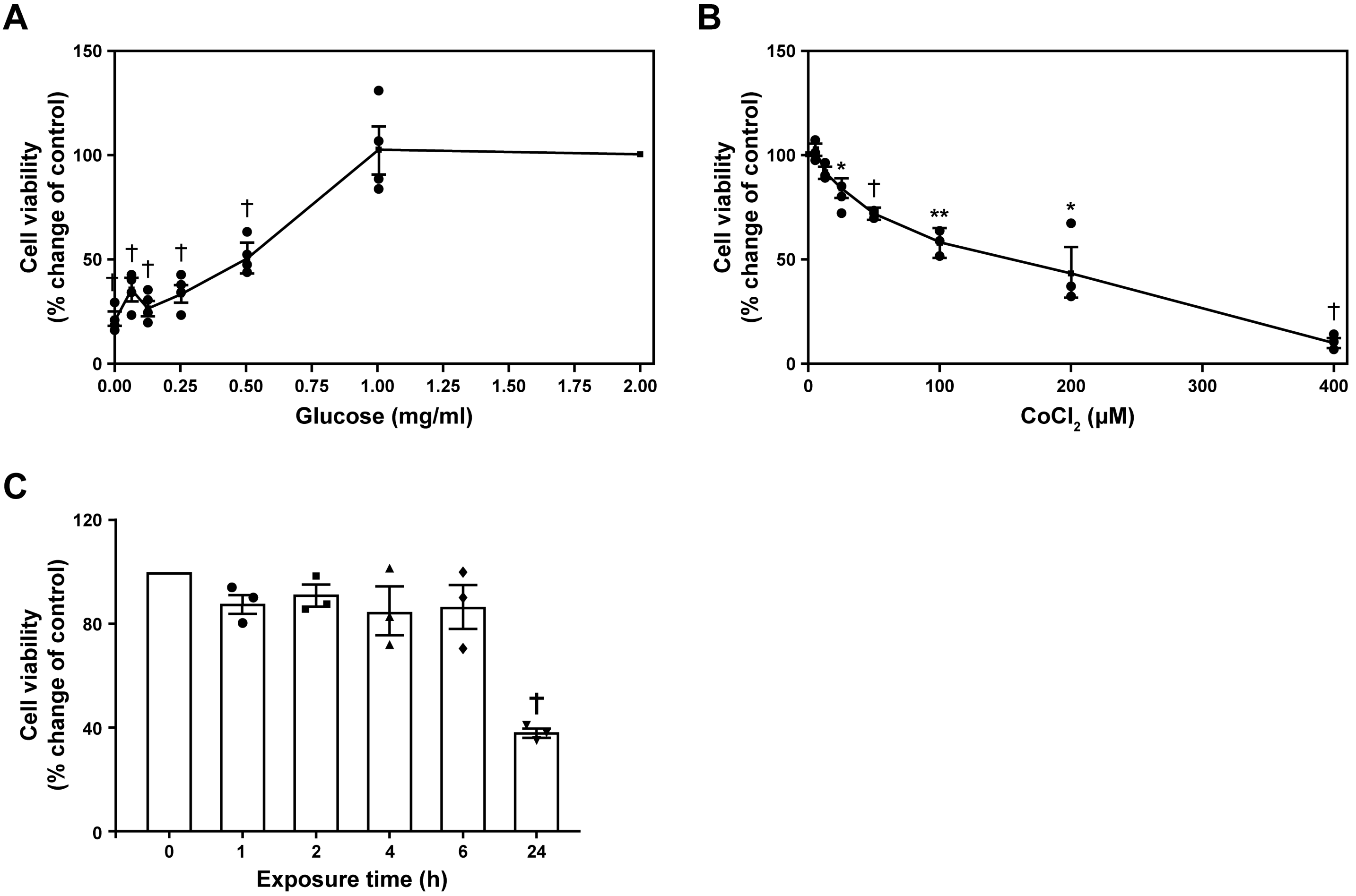

Figure S1. Effect of glucose deprivation and $CoCl_2$ treatment in viability of SH-SY5Y cells. Cell viability was determined by MTT assay. **(A)** Cells were treated with increasing concentrations of glucose (0.06-2 mg/ml) in RPMI containing 10% FBS for 24 h. Data represent mean ± S.E.M., *n*=4, † p<0.001, compared to control (2 mg/ml). **(B)** Cells were treated with increasing concentrations of $CoCl_2$ (6.25-400 μM) for 24 h. Data represent mean ± S.E.M., *n*=3, * p<0.05, ** p<0.01, † p<0.001, compared to control (0 μM). **(C)** SH-SY5Y cells were treated for 1, 2, 4, 6 and 24 hours in glucose deprivation conditions. Glucose free medium was replaced by conditioned medium, collected from exponentially growing cells, and viability was assessed at 24 h. Data represent mean ± S.E.M., *n*=3, † p<0.001, compared to control (0 h).

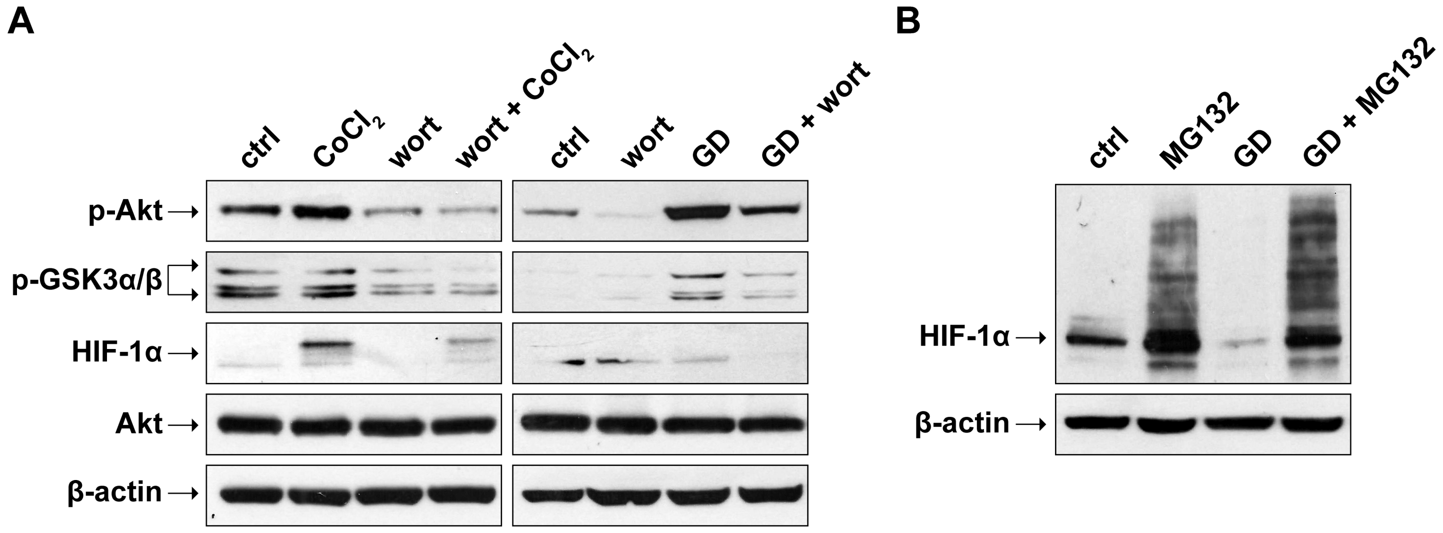

Figure S2. Effect of PI3K and proteasome inhibition on HIF-1α. **(A)** SH-SY5Y cells were pre-treated for 30 min with 100 nM wortmannin, a PI3K specific inhibitor, and incubated in complete medium with 400 μM $CoCl_2$ or in medium without glucose (glucose deprivation, GD) for 4 h. **(B)** SH-SY5Y cells were pre-treated with 10 μM MG132, a proteasome inhibitor, and incubated in medium without glucose (GD) for 4 h.

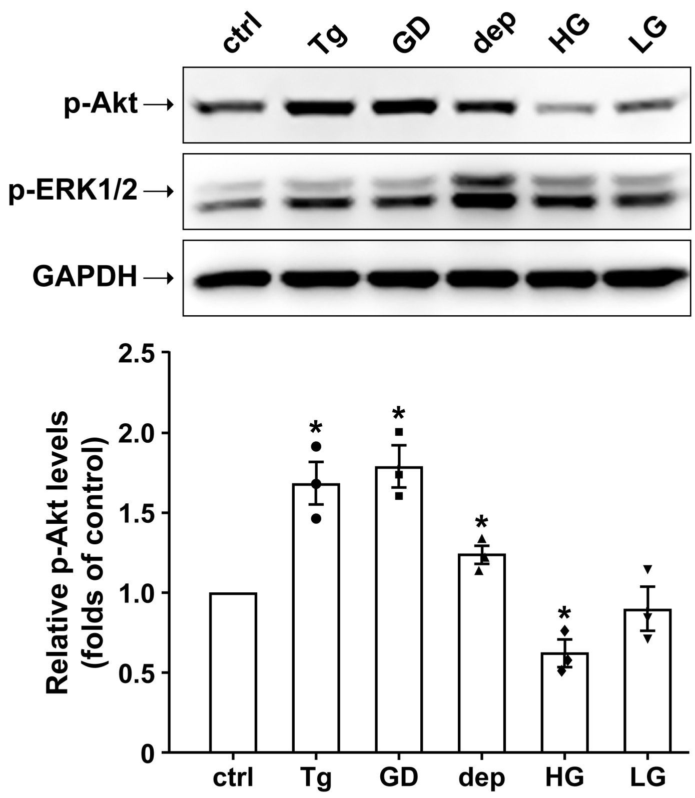

Figure S3. Akt phosphorylation in mouse primary neuronal culture. Representative western blot images and quantification of p-Akt protein levels in mouse cortical neurons. Cells were treated with Thapsigargin (Tg), Neurobasal-glucose free medium (GD), supplemented with 6 mg/ml (high glucose, HG) or 1 mg/ml (low glucose, LG) glucose for 24 h, or 60 mM KCl for 10 min in complete medium (dep). Data are presented as mean ± S.E.M., *n*=3, * $p<0.05$ compared to control cells. p-ERK1/2 were also examined as marker of depolarization.

Figure S4. Uncropped western blots.

Figure 1A

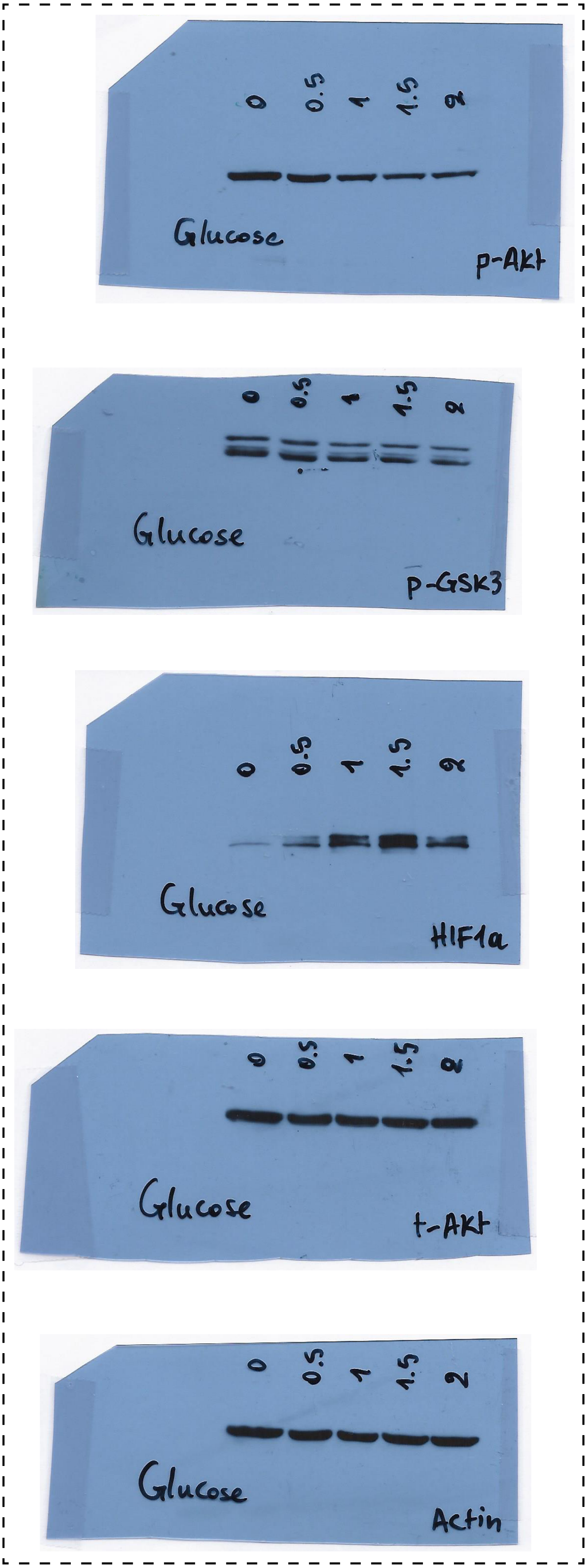

Figure 1C

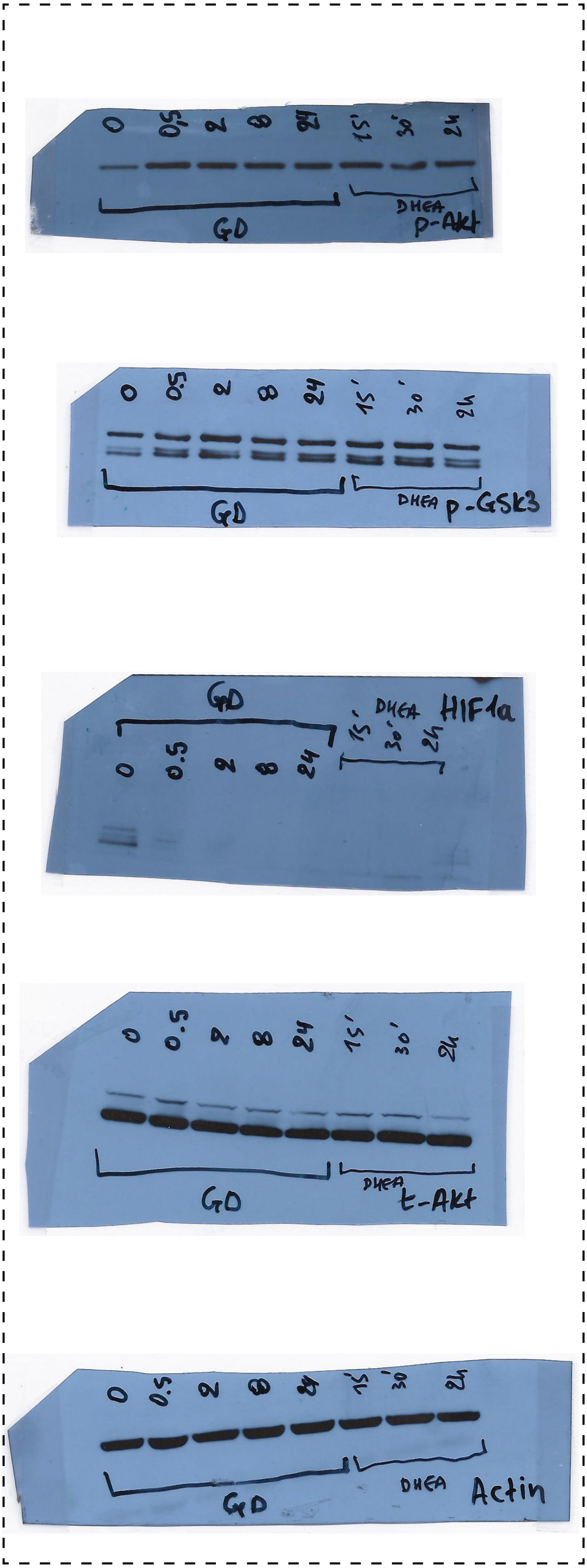

Figure 2A

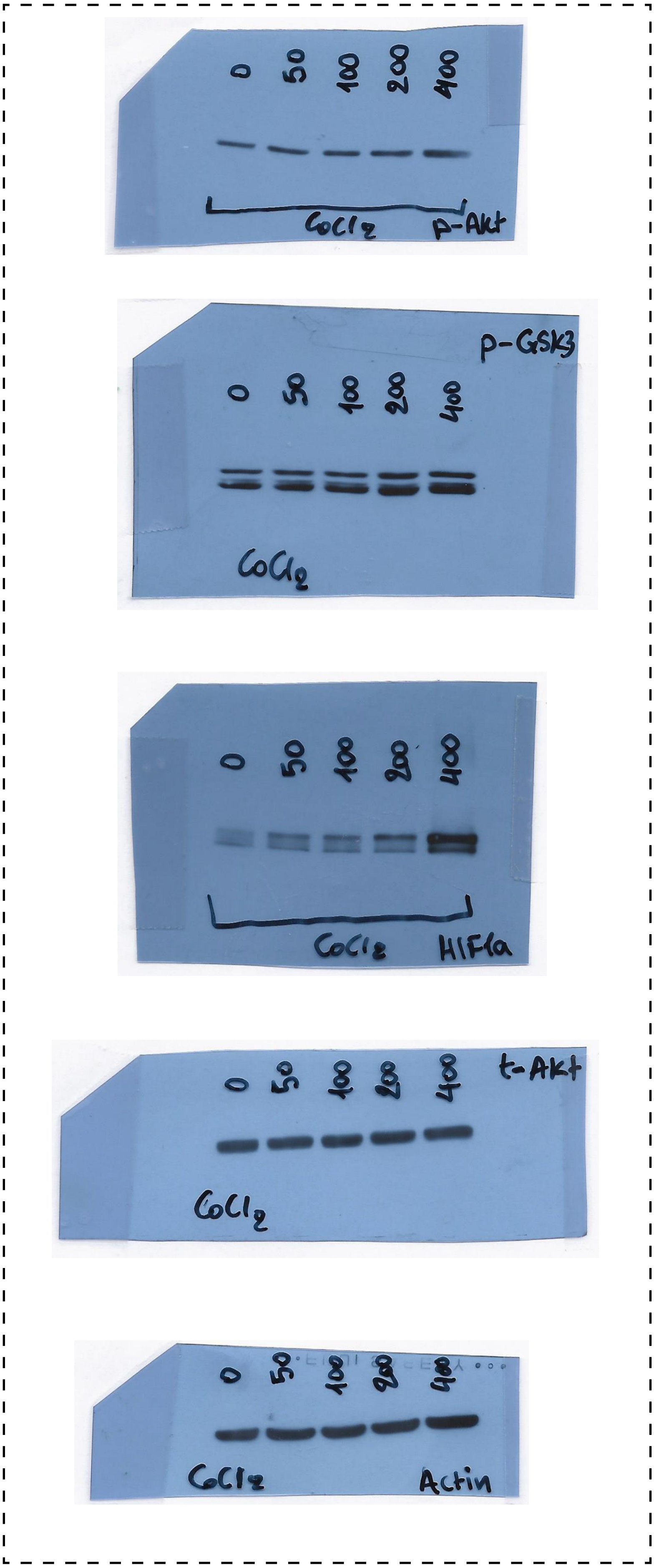

Figure 2C

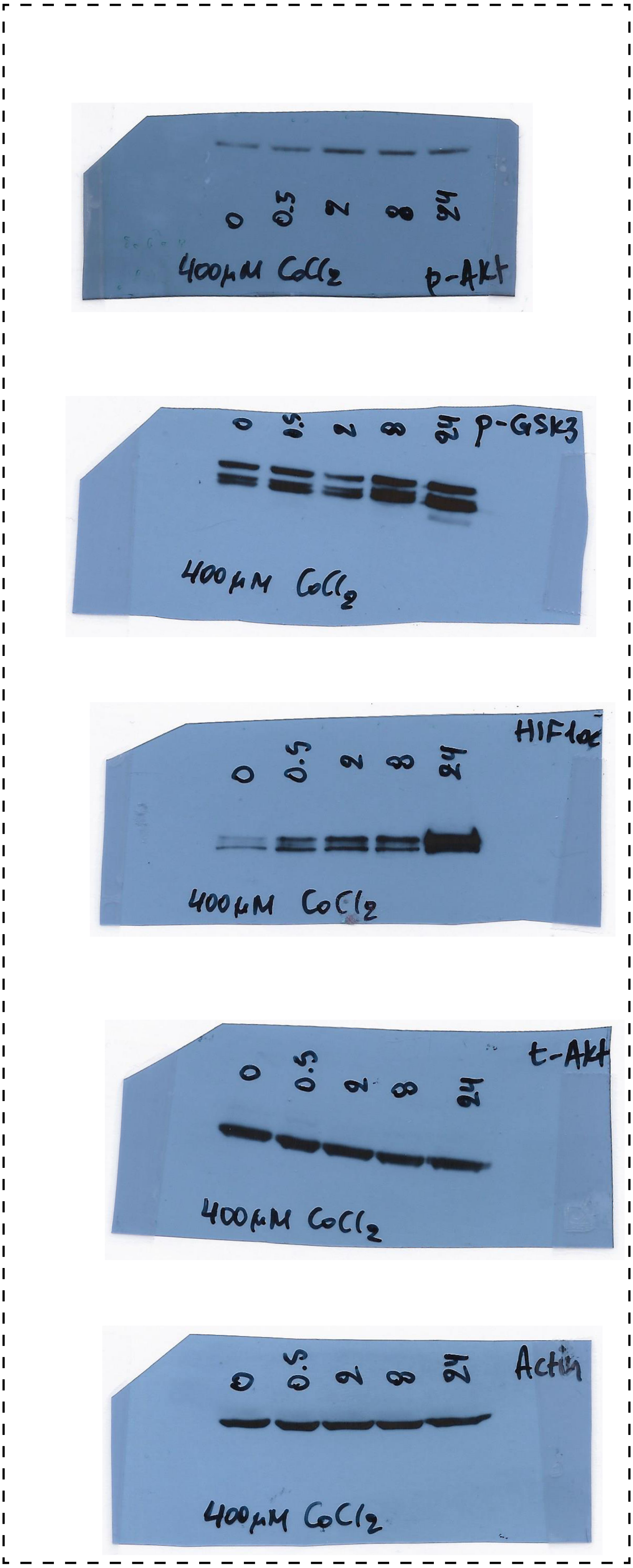

Figure 3 (2&4h)

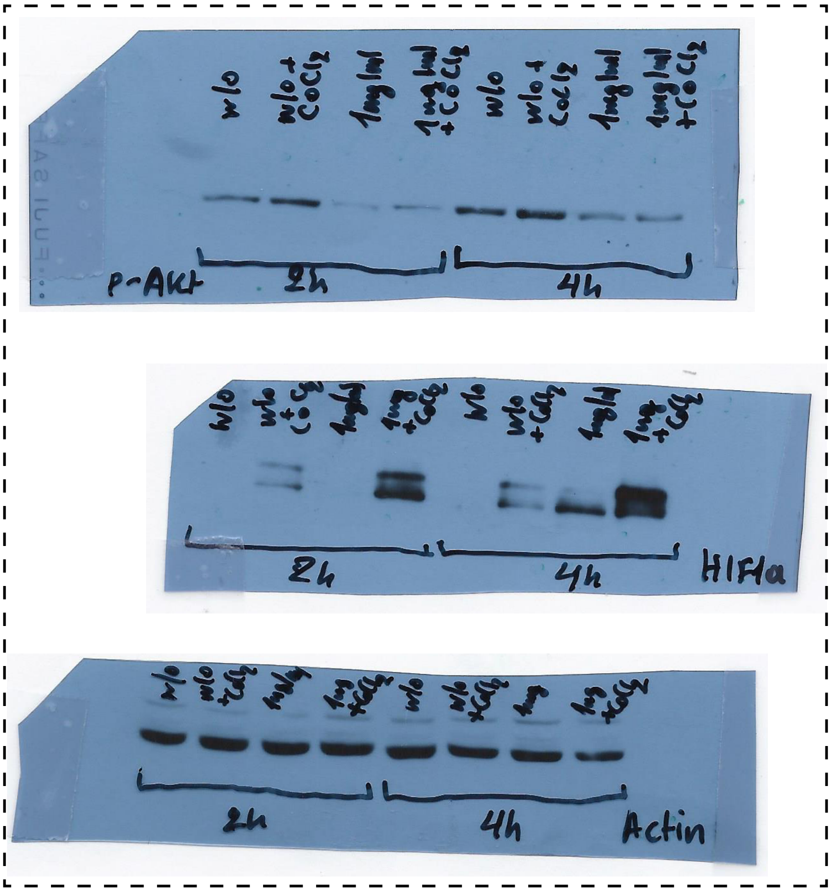

Figure 3 (24h)

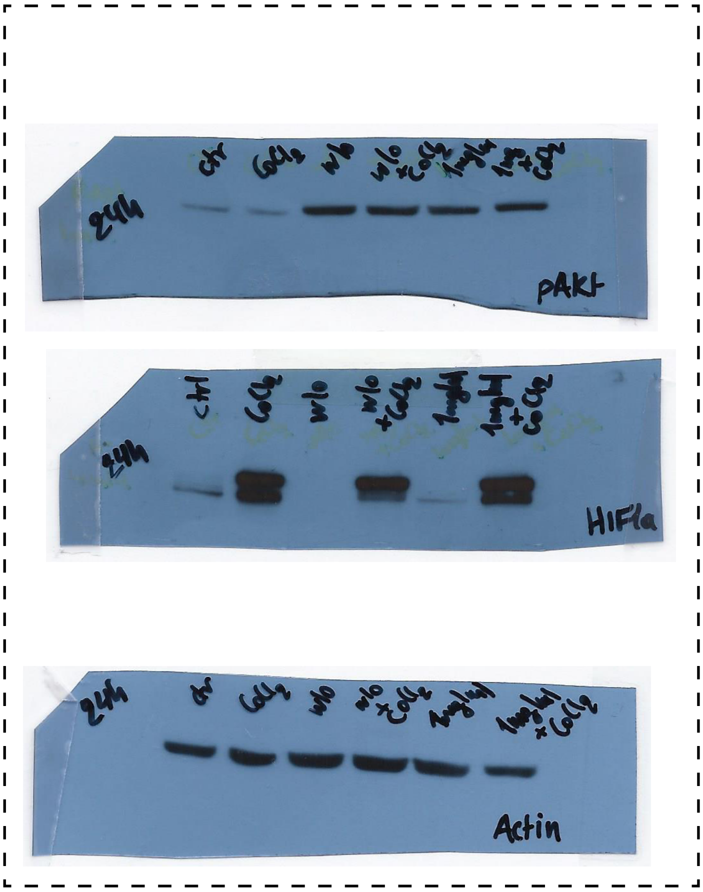

Figure 4B

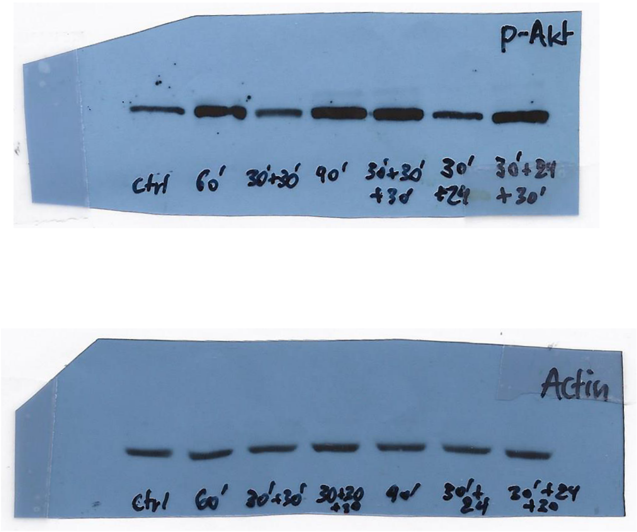

Figure 4C

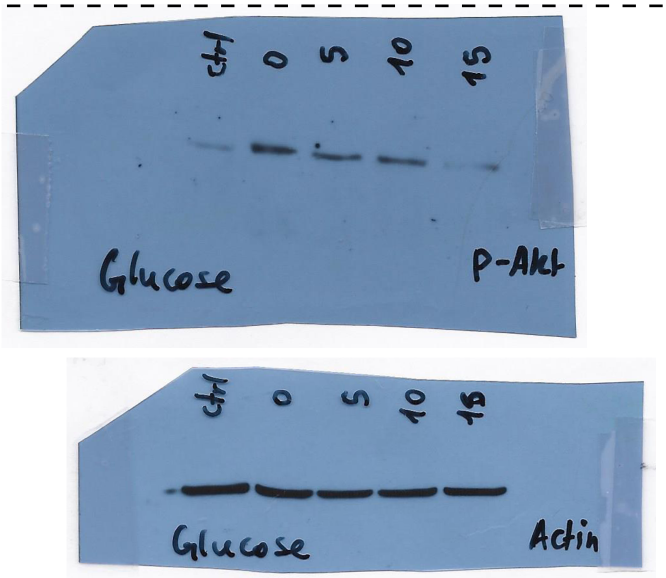

Figure 6A

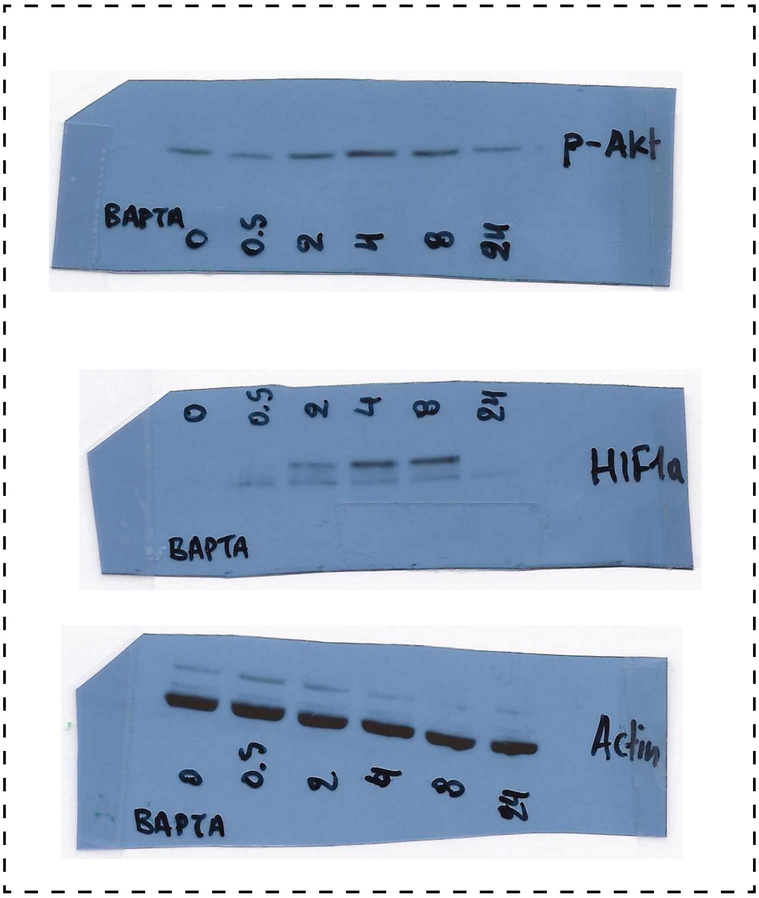

Figure 6B

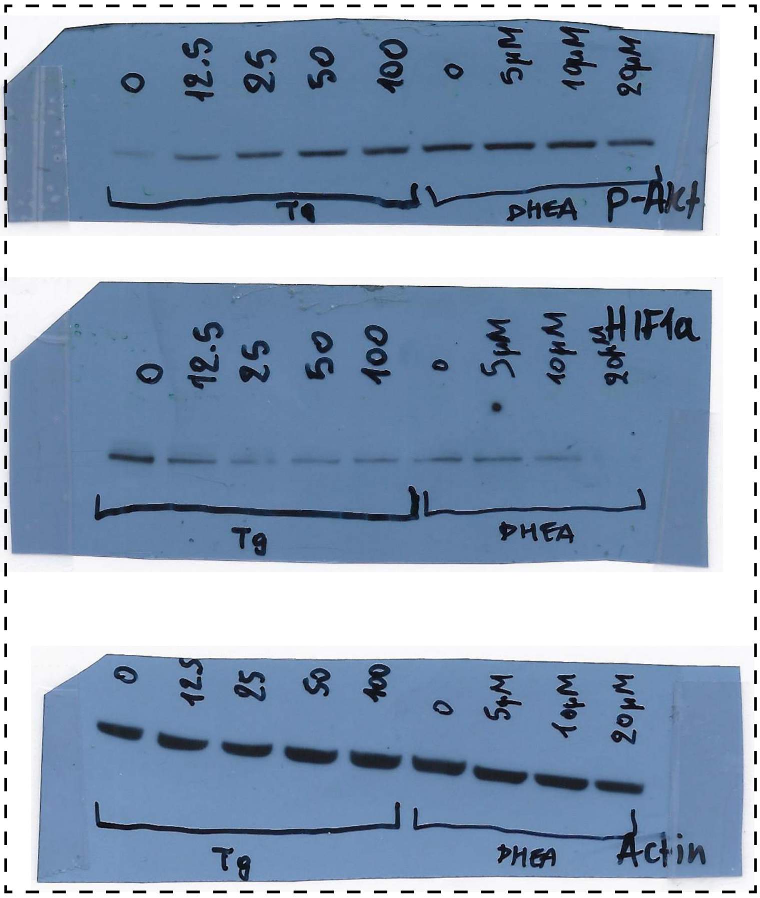

Figure 6D

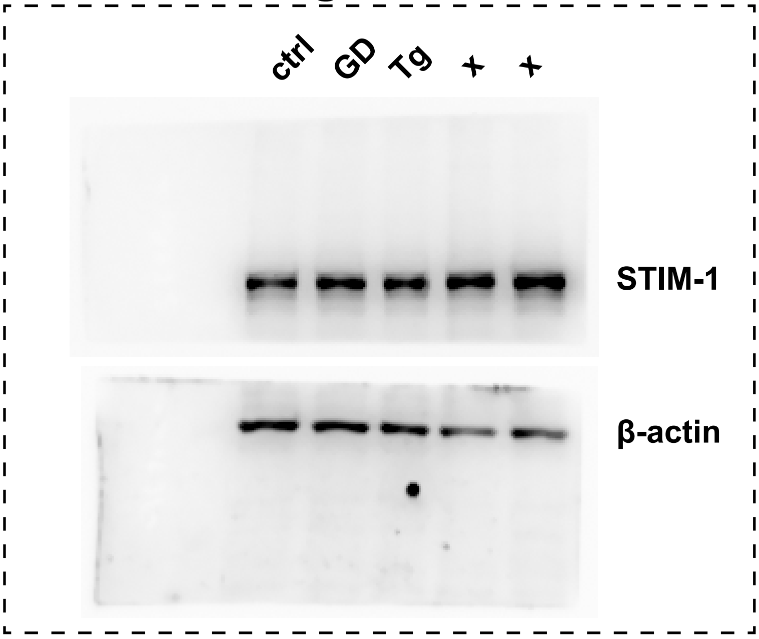

Figure 6E

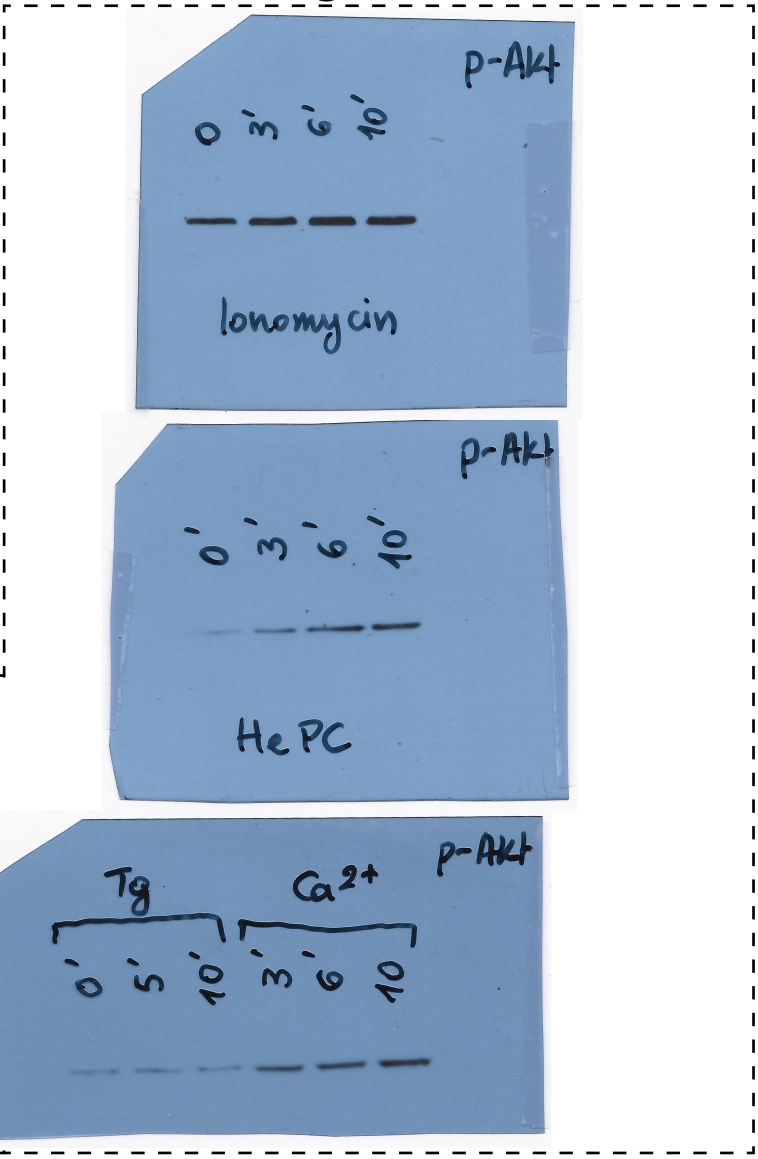

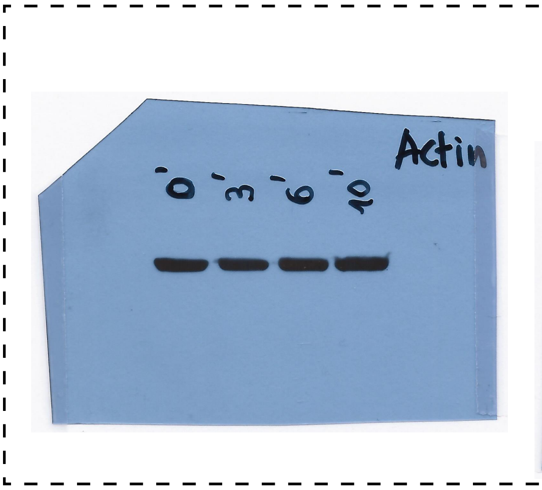

Suppl. Figure 2A ($CoCl_2$/wort)

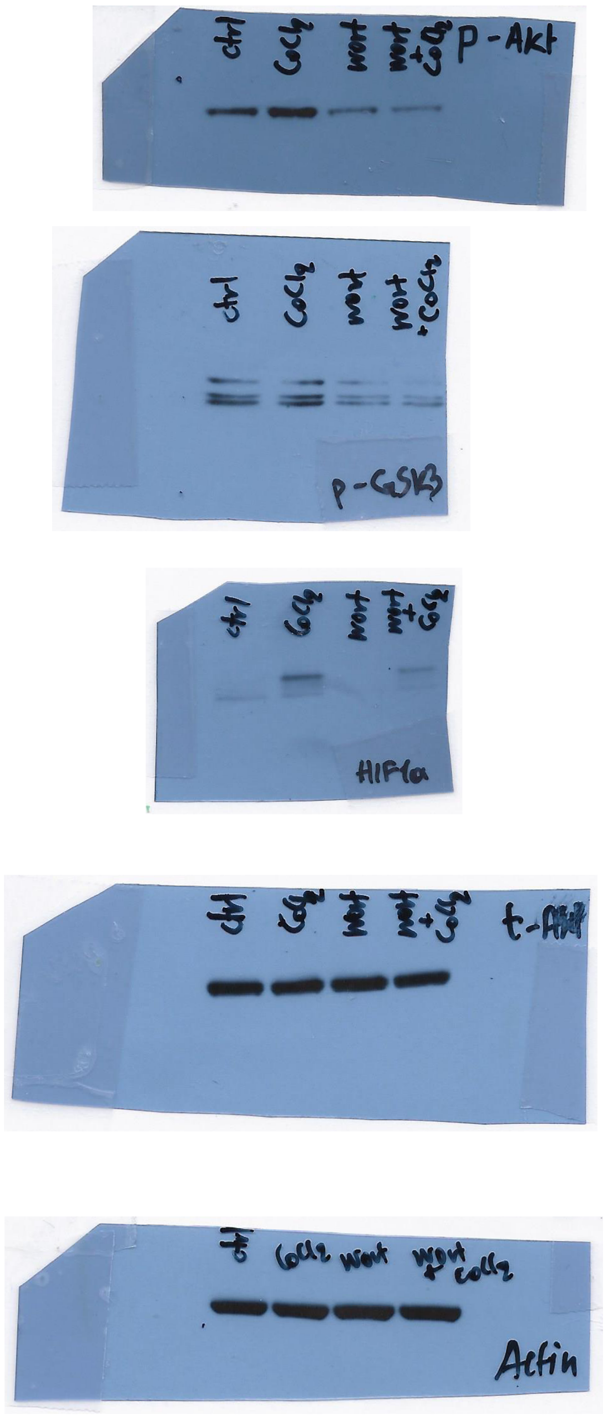

Suppl. Figure 2A (GD/wort)

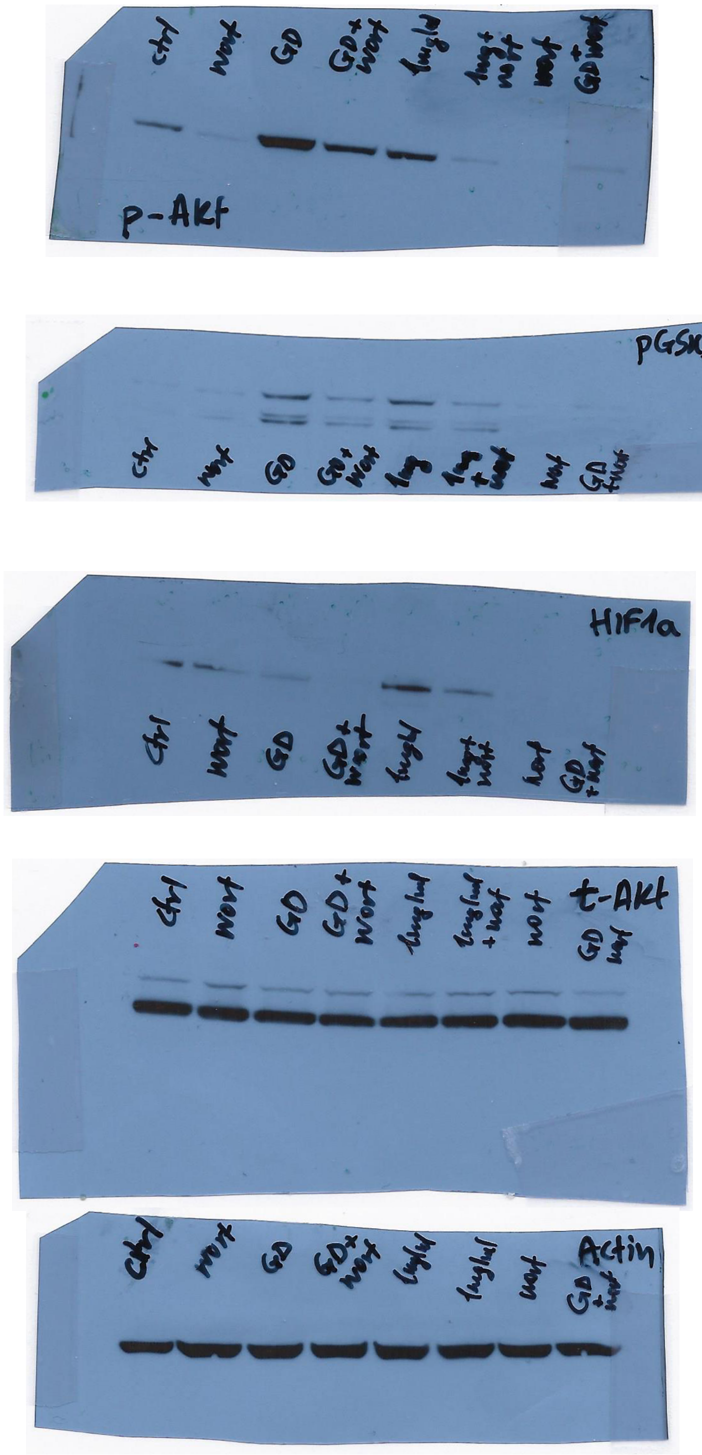

Supplementary Figure 2B

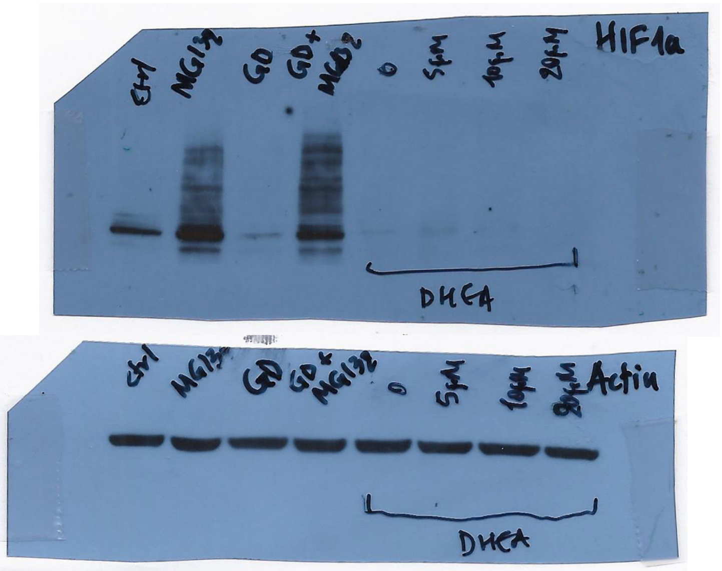

Supplementary Figure 3

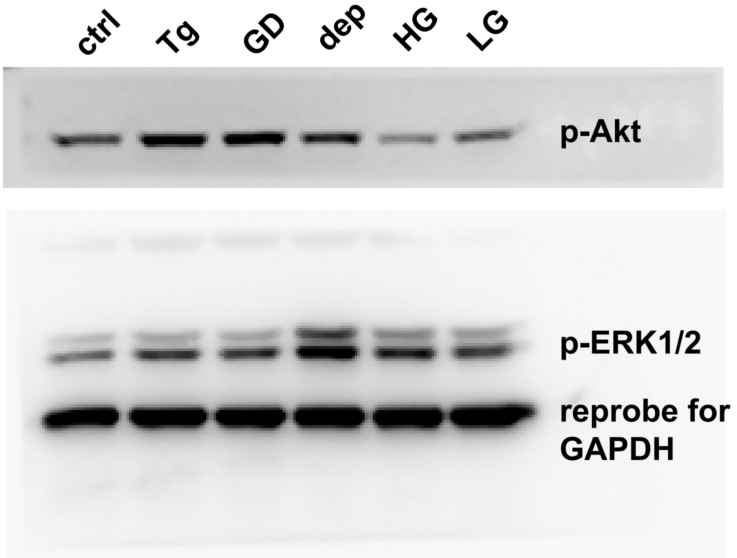

Supplement: Supplementary file 1 [file ijms-23-01386-s001.zip › ijms-1560623-supplementary.pdf]
